# Failure of Endoscopic Third Ventriculostomy

**DOI:** 10.7759/cureus.25136

**Published:** 2022-05-19

**Authors:** Jessica Lane, Syed Hassan A Akbari

**Affiliations:** 1 Department of Neurosurgery, Penn State Health Milton S. Hershey Medical Center, Hershey, USA

**Keywords:** adult hydrocephalus, hydrocephalus management, late etv failure, treatment failure, intraoperative complications, pediatric hydrocephalus, endoscopic third ventriculostomy

## Abstract

Endoscopic third ventriculostomy (ETV) is an alternative to cerebrospinal fluid (CSF) shunting in the treatment of hydrocephalus. Careful patient selection is critical as patient age, etiology of hydrocephalus, and previous shunting have been shown to influence ETV success rates. Intraoperatively, patient anatomy and medical stability may prevent or limit the completion of the ventriculostomy procedure, and findings such as a patulous third ventricular floor or cisternal scarring may portend a lower chance of successful hydrocephalus treatment. Patients in whom a ventriculostomy is completed may still experience continued symptoms of hydrocephalus or CSF leak, representing an early ETV failure. In other patients, the ETV may prove a durable treatment of hydrocephalus for several months or even years before recurrence of hydrocephalus symptoms. The failure pattern for ETV is different than that of shunting, with a higher early failure rate but improved long-term failure-free survival rates. The risk factors for failure, along with the presentation and management of failure, deserve review.

## Introduction and background

Endoscopic third ventriculostomy (ETV) can be an effective and appealing treatment for hydrocephalus; however, the procedure is not always successful. Varied success rates based on patient age, etiology of hydrocephalus, and prior shunting highlight the importance of patient selection for ETV [1,2]. A number of anatomic factors may also affect ETV success rates, including third ventricular size and shape, thickness and position of the third ventricular floor, cisternal scarring and pulsation of the ventriculostomy edges with cardiac cycle [3-5]. Operative complications that preclude the completion of the procedure may also lead to an ETV failure, and these procedures are often converted to ventricular shunting within the same operation. Perioperative failures occur in the same hospitalization as the procedure, often due to inadequate treatment of the hydrocephalus or technical issues from the procedure. Late failures occur once the patient has been discharged, sometimes after a period of months or years of clinical stability, and are generally due to closure of the stoma or cisternal scarring. This review summarizes the etiology, presentation and treatment of ETV failure based on timing of failure. Our aim is to summarize literature on the topic and provide a framework for considering ETV failure for clinicians treating hydrocephalus. 

## Review

Intraoperative failure

Patient anatomy, medical instability, and operative complications may all contribute to the inability to successfully complete an ETV. Patient selection is vital in maximizing the probability of a successful ETV. A review of imaging can identify challenging anatomy preoperatively, allowing for an adjusted surgical plan or the decision that a patient is not a good candidate for ETV. Thin cut sagittal MRI will demonstrate the shape and position of the third ventricular floor and the underlying basilar artery. Basilar artery malformation is a contraindication to the procedure. The presence of a short prepontine interval may make the procedure more challenging, but with careful planning, a successful ETV may still be possible [6]. Preoperative displacement or bowing of the third ventricular floor is a positive predictor of ETV success, with postoperative imaging often showing a reduction in displacement [5,7]. An especially thickened or patulous third ventricular floor may prove challenging operatively [8]. The size of the lateral and third ventricles, as well as the intervening foramen of Monro, will affect the ease of the procedure. Slit ventricles or stenotic/obstructed foramen of Monro will make endovascular navigation challenging and the chance of intraoperative injury more likely. Patients with myelomeningocele often have ventricular abnormalities, which can negatively impact the surgeon’s orientation and ability to perform the third ventriculostomy. A review of 455 patients with hydrocephalus secondary to myelomeningocele found third ventricular abnormalities in 41% of patients, with the most common being prominent massa intermedia, narrow tuber cinereum, thickened or steep or vascular third ventricular floor, and interhypothalamic or mamillary adhesions [9]. Patients with a history of hemorrhage or severe infection may also have distorted anatomy that should be well studied with imaging before attempting an ETV.

While a close review of patient demographics and imaging preoperatively helps to select appropriate patients for ETV, endoscopic visualization may reveal an inhospitable corridor for ventriculostomy. In this situation, the lamina terminalis may be inspected as an alternate point of fenestration if the endoscopic trajectory and view allow. The visualization itself may be the limiting factor due to debris within the cerebrospinal fluid, such as from tumor, infection, or hemorrhage, and cause the procedure to be aborted. The surgeon may successfully create an opening in the floor of the third ventricle, only to struggle to appropriately enlarge this due to thickening or scarring. She may also create a satisfactory ostomy only to discover scarring or adhesions within the membrane of Lillequist that are unable to be lysed. Rates of intraoperative failure can vary with the etiology of hydrocephalus, availability of preoperative imaging, and experience of the surgical team. In a comparison of ETV in Uganda compared to pooled data from Canada, Israel, and the UK, there was a 29% rate of intraoperative failure in the Ugandan cohort versus 2% in the comparison cohort [10]. The authors note that the median Endoscopic Third Ventriculostomy Success Score (ETVSS) was substantially lower in the Ugandan cohort and that the lack of preoperative MRI leads to attempted ETV in patients with unfavorable anatomy who might not have been offered the procedure in other settings. A single-center series of children and young adults in the United States noted a procedure abandonment rate of 26%, with prior shunting leading to a greater likelihood of abandonment [8]. A large meta-analysis including both adult and pediatric patients found an overall rate of procedure abandonment at 4.2% [11].

While performing the ETV, it may be possible to injure one of the smaller vessels in and around the ventricular system. This may cloud the view and render an endoscopic approach impossible. Usually, bleeding from these smaller vessels may be stopped with the use of gentle irrigation or tamponade from an instrument or the endoscope itself. The more catastrophic possibility of a basilar artery injury will usually result in the aborting of an ETV and the need for possible interventional procedures [12]. Basilar artery rupture is rare and reported in 0.2% of ETVs, but rates of all intraoperative bleeding range from 0%-8.5%, with less than 1% of ETV procedures being abandoned due to hemorrhage [13].

Cardiorespiratory concerns from the anesthesia team are, as with all surgery, a possible reason to abort a planned procedure. In ETV specifically, bradycardia can be seen with the creation and enlargement of the ventriculostomy or with the need to enlarge the ventricles with irrigation to allow for endoscopic navigation. While this will often resolve with a pause in manipulation, continued bradycardia needs to be addressed. Rates of bradycardia related to ventriculostomy have been cited between 6%-27%, and there are reported asystolic events [14,15]. Symptoms of increased intracranial pressure, including bradycardia, can also be seen when irrigation used for the procedure is not allowed to drain from the ventricles, which may be addressed with open endoscope ports, aspiration of fluid through the working channels of the endoscope, or withdrawal of the endoscope allowing fluid to drain through the sheath [16]. Unfortunately, herniation has been reported in instances where irrigation was passed through an inadvertently closed endoscope system [8,17].

When the decision is made to abort the procedure, a number of options remain. A ventricular shunt may be placed within the same procedure. For this reason, many surgeons will position patients undergoing an ETV as they would a ventriculoperitoneal shunt insertion in the event a shunt needs to be placed during the operation. Alternatively, if the surgeon believes the ETV may yet be successful at a later date, an external ventricular drain (EVD) or tapping reservoir may be left in place to temporize the patient until an ETV may be reattempted. An EVD has the added benefit of being able to monitor intracranial pressures in the perioperative phase. Some surgeons advocate leaving the drain clamped to monitor pressure, allowing for a higher than normal threshold for opening the drain, with the hope that the added pressure might keep the ostomy patent [18,19].

Perioperative failure

Failure of an ETV after its creation can occur because the ETV does not adequately treat the hydrocephalus or because the new stoma ceases to function. In cases where the failure becomes evident immediately after surgery, without a period of improvement, likely, the ETV was not sufficient to treat the hydrocephalus during the index procedure [20]. CSF absorption may be poor, in which case patients would be expected to present soon after ETV with continued signs and symptoms of elevated intracranial pressure while imaging and repeat endoscopy would reveal an open stoma and flow of CSF [21]. Alternatively, the ETV may not function due to membranes or adhesions beneath the floor of the third ventricle, which were missed and left intact during the initial surgery [21]. Scarring of the prepontine cistern has been shown in multiple cohorts to be a negative prognostic factor for ETV success [4,22,23].

The presentation of ETV failure in these situations will often reflect the signs and symptoms of initial presentation, for example, a bulging fontanelle or Parinaud’s phenomenon in neonates. The continued elevation of intracranial pressure in the setting of a new tract to the subgaleal surface may also cause a cerebrospinal fluid leak through the incision should an ETV fail despite adequate technical closure. Additionally, in those patients in whom a ventricular drainage catheter has been left in place, high output or elevated intracranial pressure (ICP) readings may indicate failure. It is worth noting that many authors have noted a period of adaptation a few days after ventriculostomy, during which intracranial pressures may be transiently elevated [18,24]. This is often managed with some permissive hypertension in the absence of symptomatology, as the additional pressure may theoretically help keep the ETV patent, or intermittent CSF removal for a few days before declaring ETV failure [19,25]. Should a drain need to be opened, it may be prudent to attempt a delayed clamp trial before declaring true ETV failure and proceeding with shunt placement.

In cases of perioperative ETV failure, imaging should be performed to rule out an operative complication, for example, intraventricular hemorrhage obstructing the stoma [26]. Reduction in ventriculomegaly is not always obvious in early postoperative imaging and should not be seen as requisite for a functional ETV [27]. While the appearance of a flow void on MRI and use of cine phase-contrast MRI is somewhat correlated with ETV success, this is mostly studied at a later stage of follow-up, and the absence of these imaging findings in the immediate postoperative setting is not diagnostic for ETV failure [16,27-29]. Repeat ventriculostomy may be performed if there is concern for unfenestrated membranes, inadequately sized ventriculostomy, or other treatable pathology. Alternately, a shunt may be placed. ETV failure or aborted ETV procedures have not been shown to increase the risk of failure or infection in subsequently placed shunts [30].

Late ETV failure

Predicting the failure of an ETV after the acute operative/perioperative period has been subject to significant study in the literature. Patient selection factors may influence the risk of ETV failure weeks to years after surgery. Age appears to be a strong predictor of ETV success, with infants less than six months old having the highest risk of failure, in some cases up to a five-fold increase in risk compared to older patients [1,31-33]. It has been suggested that the milieu of growth factors in the CSF predisposes young infants to the closure of the ventriculostomy [16,34]. Additionally, young infants are thought to have a lower capacity for CSF absorption secondary to immature arachnoid granulations, which require a higher pressure gradient for absorption [33,35]. The importance of the functional absorption of CSF from the subarachnoid space also explains why etiology affects ETV success. Hydrocephalus caused by hemorrhage or infection, both of which can cause dysfunction of the subarachnoid space, are less likely to be successfully treated with ETV compared to hydrocephalus caused by aqueductal stenosis or tectal tumors. Similarly, patients with previous shunting, which is thought to decrease absorptive capability, are more prone to ETV failure [16]. These risk factors for failure may be related, as the etiologies for hydrocephalus differ in older and younger children, and rates of previous shunting may be related to etiology. These three risk factors have been combined into an Endoscopic Third Ventriculostomy Success Score (ETVSS, Table 1), which was designed to predict ETV success at six months postoperatively, but has since been validated in multiple settings and for longer-term outcomes [1,2,36-39]. In the ETVSS, the score represented the predicted percentage of successful cases. For instance, a three-month-old child with postinfectious hydrocephalus and no previous shunt would have an ETVSS of 20 and a 20% predicted success rate, compared to a 12-year-old with a tectal tumor and no previous shunt with a predicted success rate of 90%.

**Table 1:** ETV Success Score The ETV Success Score, as described in Kulkarni et al., uses patient age, hydrocephalus etiology, and history of shunting to predict the likelihood of successful ETV. ETV Success Score, from Kulkarni et al. [1]

Score =	Age +	Etiology +	Previous shunt
0	<1 month	Postinfectious	Previous shunt
10	1 month to <6 months		No previous shunt
20		Myelomeningocele, intraventricular hemorrhage, non-tectal brain tumor	
30	6 months to 1 year	Aqueductal stenosis, tectal tumor, other	
40	1 year to <10 years		
50	10 years or older		

For excellent ETV candidates with ETVSS of 80 or above, the risk of ETV failure appears to be lower than the risk of shunt failure from the beginning, becoming even more favorable with time. In patients with ETVSS 70 or below, the initial risk of ETV failure exceeds that of shunt failure in comparable patients but becomes lower than shunt failure risk by 3-6 months postoperatively [40].

**Figure 1:**
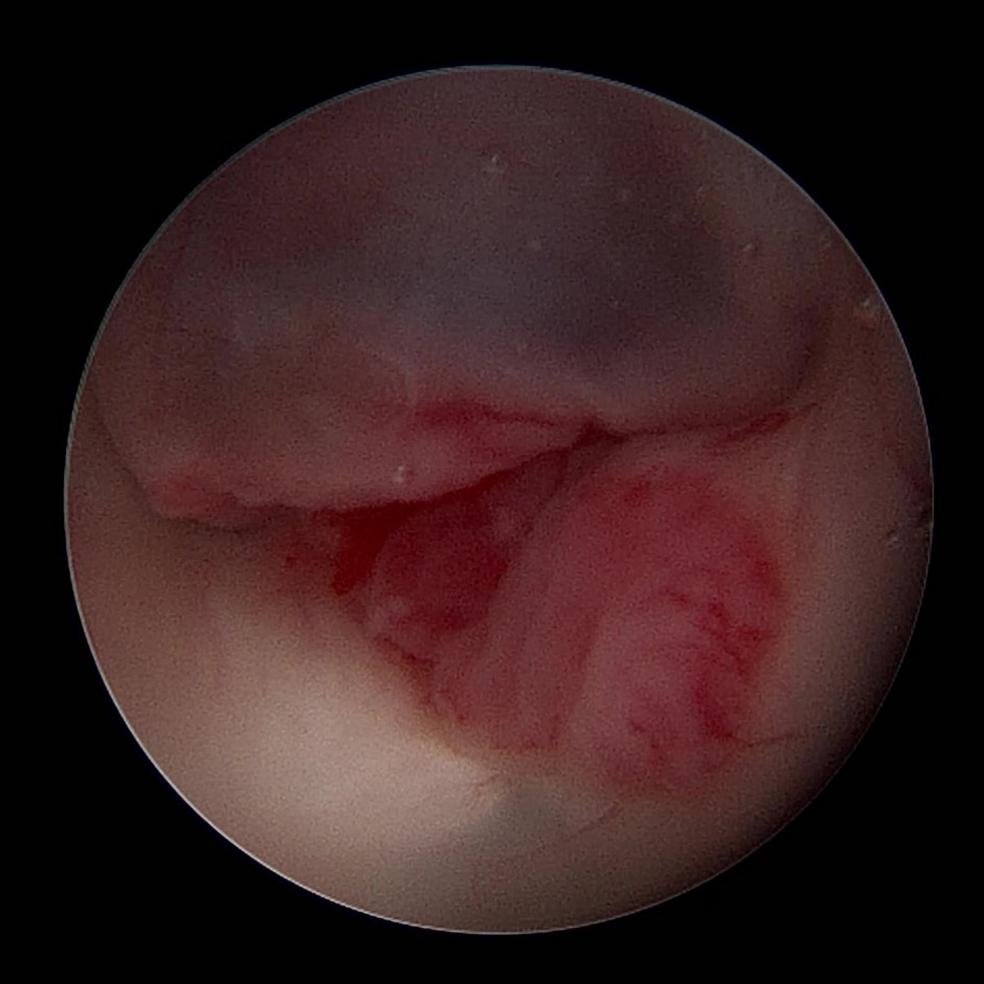
Patulous floor of third ventricle with tumor infiltration. An endoscopic image of a patulous third ventricular floor in the authors' patient undergoing ETV with a glial tumor.

Even in the well-selected patient, ETVs may fail due to technical issues from the initial surgery. For example, a smaller ETV may be more likely to scar, as is an ostomy that was created using monopolar cautery [41,42]. The charred edges of an ETV performed in this manner are more likely to cause scarring and ETV failure. ETVs that are particularly bloody may undergo the same issues, as the blood clot can either plug the ostomy or promote inflammatory reactions that will promote scar formation [28,43]. Debris from tumors may also cause closure of the ETV [26,44]. Stenting of the ventriculostomy has been reported in cases where the risk of restenosis was felt to be high due to tumor or redundant tissue from the third ventricular floor (Figure 1) [45,46].

Usually, if a patient initially tolerates an ETV and fails in a delayed fashion, this is due to the reclosure of the stoma. The stoma itself may close or stenosis due to scarring or gliosis, causing the floor of the third ventricle to appear intact either by ventriculoscopy or imaging. There can be a second membrane beneath the floor of the third ventricle or redundancy of the membrane of Lilliquist that blocks the flow of CSF from the third ventricle to the prepontine cistern, despite an open stoma. Arachnoid webbing may also develop within the cistern, blocking absorption [20,21,47,48]. Evidence of stoma closure may be apparent on MRI imaging, especially with flow-sensitive modalities [26-28,47]. In a mixed-age cohort, 14 symptomatic patients underwent cine phase-contrast MRI before endoscopic re-exploration; the stoma obstruction was confirmed in all 10 patients with no flow on MRI, and patency was confirmed in the remaining four with the flow on their imaging [28]. In another series of 67 pediatric patients, surveillance cine phase-contrast MRI identified stoma obstruction in five asymptomatic patients greater than a year after ETV and after multiple earlier images demonstrated flow through the stoma [49]. However, there are series where the detection of flow was not well associated with clinical outcomes, such as an American series of 89 patients where 23% of patients with good clinical outcomes from ETV had no imaging evidence of flow through the stoma while there was the appearance of flow in 58% of the clinical failures [3].

As these mechanisms of stoma closure require time, the expected clinical course would be a period of improvement after the initial ETV followed by late presenting symptoms of ICP elevation [21]. When a ventriculostomy fails due to closure of the stoma, a repeat ventriculostomy may be attempted. Success rates for reopening a closed ETV vary greatly, 37%-78% in published series [20,47,48,50,51]. A long time from initial surgery to failure is associated with the increased success of the repeat operation, with an interval greater than six months associated with 90% success in one series [20]. Lack of subarachnoid adhesions is associated with higher rates of success, but the effect of patient age at surgery is conflicting in the literature [20,50,51].

One of the benefits of ETV over shunting, especially in regions where resources are limited, is that the long-term failure rate is low compared to shunts, despite a higher rate of failure in the early postoperative period. In a propensity score-adjusted analysis, Kulkarni and colleagues demonstrate that while the early risk of failure is at least 20% greater in ETV compared to shunting, after three months this relative risk decreases steadily, and the risk of ETV failure at two years is half the risk of shunt failure [52]. While rare, very late failure after ETV has been reported after years of clinical stability, and in some of these cases, the clinical deterioration is rapid with very poor outcomes [53]. Patients should be counseled about this possibility and the importance of urgent medical treatment in the setting of failure symptoms.

## Conclusions

ETV can be a safe and effective treatment for hydrocephalus in well-selected patients. Nevertheless, several risk factors make the procedure more difficult to successfully complete and more likely to fail over time. While generally, the risk of ETV failure is the greatest in the perioperative and early postoperative period, there are also cases of failure after years of clinical stability. Surgeons should assess a patient’s preoperative risk factors based on history and imaging before attempting an ETV and should also use these and intraoperative risk factors in planning follow-up and possible repeat procedures.

## References

[1] Kulkarni AV, Drake JM, Mallucci CL, Sgouros S, Roth J, Constantini S (2009). Endoscopic third ventriculostomy in the treatment of childhood hydrocephalus. J Pediatr.

[2] Kulkarni AV, Riva-Cambrin J, Browd SR (2011). Use of the ETV Success Score to explain the variation in reported endoscopic third ventriculostomy success rates among published case series of childhood hydrocephalus. J Neurosurg Pediatr.

[3] Greenfield JP, Hoffman C, Kuo E, Christos PJ, Souweidane MM (2008). Intraoperative assessment of endoscopic third ventriculostomy success. J Neurosurg Pediatr.

[4] Warf BC, Campbell JW, Riddle E (2011). Initial experience with combined endoscopic third ventriculostomy and choroid plexus cauterization for post-hemorrhagic hydrocephalus of prematurity: the importance of prepontine cistern status and the predictive value of FIESTA MRI imaging. Childs Nerv Syst.

[5] Dlouhy BJ, Capuano AW, Madhavan K, Torner JC, Greenlee JD (2012). Preoperative third ventricular bowing as a predictor of endoscopic third ventriculostomy success. J Neurosurg Pediatr.

[6] Souweidane MM, Morgenstern PF, Kang S, Tsiouris AJ, Roth J (2010). Endoscopic third ventriculostomy in patients with a diminished prepontine interval. J Neurosurg Pediatr.

[7] Foroughi M, Wong A, Steinbok P, Singhal A, Sargent MA, Cochrane DD (2011). Third ventricular shape: a predictor of endoscopic third ventriculostomy success in pediatric patients. J Neurosurg Pediatr.

[8] Brockmeyer D, Abtin K, Carey L, Walker ML (1998). Endoscopic third ventriculostomy: an outcome analysis. Pediatr Neurosurg.

[9] Etus V, Guler TM, Karabagli H (2017). Third ventricle floor variations and abnormalities in myelomeningocele-associated hydrocephalus: our experience with 455 endoscopic third ventriculostomy procedures. Turk Neurosurg.

[10] Kulkarni AV, Warf BC, Drake JM, Mallucci CL, Sgouros S, Constantini S (2010). Surgery for hydrocephalus in sub-Saharan Africa versus developed nations: a risk-adjusted comparison of outcome. Childs Nerv Syst.

[11] Bouras T, Sgouros S (2011). Complications of endoscopic third ventriculostomy. J Neurosurg Pediatr.

[12] Abtin K, Thompson BG, Walker ML (1998). Basilar artery perforation as a complication of endoscopic third ventriculostomy. Pediatr Neurosurg.

[13] Bouras T, Sgouros S (2013). Complications of endoscopic third ventriculostomy. World Neurosurg.

[14] Kawsar KA, Haque MR, Chowdhury FH (2015). Avoidance and management of perioperative complications of endoscopic third ventriculostomy: the Dhaka experience. J Neurosurg.

[15] Baykan N, Isbir O, Gerçek A, Dağçnar A, Ozek MM (2005). Ten years of experience with pediatric neuroendoscopic third ventriculostomy: features and perioperative complications of 210 cases. J Neurosurg Anesthesiol.

[16] Douglas Hardesty and Andrew S. Little (2016). Endoscopic third ventriculostomy. Neuroendoscopic Surgery.

[17] Schroeder HW, Niendorf WR, Gaab MR (2002). Complications of endoscopic third ventriculostomy. J Neurosurg.

[18] Bellotti A, Rapanà A, Iaccarino C, Schonauer M (2001). Intracranial pressure monitoring after endoscopic third ventriculostomy: an effective method to manage the 'adaptation period'. Clin Neurol Neurosurg.

[19] Muroi A, Quezada JJ, McComb JG (2021). Usefulness of postoperative ventriculography and intracranial pressure monitoring following endoscopic third ventriculostomy. Childs Nerv Syst.

[20] Marano PJ, Stone SS, Mugamba J, Ssenyonga P, Warf EB, Warf BC (2015). Reopening of an obstructed third ventriculostomy: long-term success and factors affecting outcome in 215 infants. J Neurosurg Pediatr.

[21] Wagner W, Koch D (2005). Mechanisms of failure after endoscopic third ventriculostomy in young infants. J Neurosurg.

[22] Warf BC, Kulkarni AV (2010). Intraoperative assessment of cerebral aqueduct patency and cisternal scarring: impact on success of endoscopic third ventriculostomy in 403 African children. J Neurosurg Pediatr.

[23] Chamiraju P, Bhatia S, Sandberg DI, Ragheb J (2014). Endoscopic third ventriculostomy and choroid plexus cauterization in posthemorrhagic hydrocephalus of prematurity. J Neurosurg Pediatr.

[24] Hopf NJ, Grunert P, Fries G, Resch KD, Perneczky A (1999). Endoscopic third ventriculostomy: outcome analysis of 100 consecutive procedures. Neurosurgery.

[25] Cinalli G, Spennato P, Ruggiero C (2006). Intracranial pressure monitoring and lumbar puncture after endoscopic third ventriculostomy in children. Neurosurgery.

[26] Fukuhara T, Vorster SJ, Ruggieri P, Luciano MG (1999). Third ventriculostomy patency: comparison of findings at cine phase-contrast MR imaging and at direct exploration. AJNR Am J Neuroradiol.

[27] Kulkarni AV, Drake JM, Armstrong DC, Dirks PB (2000). Imaging correlates of successful endoscopic third ventriculostomy. J Neurosurg.

[28] Fukuhara T, Luciano MG, Kowalski RJ (2002). Clinical features of third ventriculostomy failures classified by fenestration patency. Surg Neurol.

[29] Kunz M, Schulte-Altedorneburg G, Uhl E, Schmid-Elsaesser R, Schöller K, Zausinger S (2008). Three-dimensional constructive interference in steady-state magnetic resonance imaging in obstructive hydrocephalus: relevance for endoscopic third ventriculostomy and clinical results. J Neurosurg.

[30] Warf BC, Bhai S, Kulkarni AV, Mugamba J (2012). Shunt survival after failed endoscopic treatment of hydrocephalus. J Neurosurg Pediatr.

[31] Warf BC, Mugamba J, Kulkarni AV (2010). Endoscopic third ventriculostomy in the treatment of childhood hydrocephalus in Uganda: report of a scoring system that predicts success. J Neurosurg Pediatr.

[32] Sacko O, Boetto S, Lauwers-Cances V, Dupuy M, Roux FE (2010). Endoscopic third ventriculostomy: outcome analysis in 368 procedures. J Neurosurg Pediatr.

[33] Drake JM (2007). Endoscopic third ventriculostomy in pediatric patients: the Canadian experience. Neurosurgery.

[34] Fritsch MJ, Kienke S, Ankermann T, Padoin M, Mehdorn HM (2005). Endoscopic third ventriculostomy in infants. J Neurosurg.

[35] Koch D, Wagner W (2004). Endoscopic third ventriculostomy in infants of less than 1 year of age: which factors influence the outcome?. Childs Nerv Syst.

[36] Furtado LM, da Costa Val Filho JA, Dos Santos Júnior EC (2021). External validation of the ETV success score in 313 pediatric patients: a Brazilian single-center study. Neurosurg Rev.

[37] Naftel RP, Reed GT, Kulkarni AV, Wellons JC (2011). Evaluating the Children's Hospital of Alabama endoscopic third ventriculostomy experience using the endoscopic third ventriculostomy success score: an external validation study. J Neurosurg Pediatr.

[38] Durnford AJ, Kirkham FJ, Mathad N, Sparrow OC (2011). Endoscopic third ventriculostomy in the treatment of childhood hydrocephalus: validation of a success score that predicts long-term outcome. J Neurosurg Pediatr.

[39] Breimer GE, Dammers R, Woerdeman PA, Buis DR, Delye H, Brusse-Keizer M, Hoving EW (2017). Endoscopic third ventriculostomy and repeat endoscopic third ventriculostomy in pediatric patients: the Dutch experience. J Neurosurg Pediatr.

[40] Kulkarni AV, Drake JM, Kestle JR, Mallucci CL, Sgouros S, Constantini S (2010). Predicting who will benefit from endoscopic third ventriculostomy compared with shunt insertion in childhood hydrocephalus using the ETV Success Score. J Neurosurg Pediatr.

[41] Kombogiorgas D, Sgouros S (2006). Assessment of the influence of operative factors in the success of endoscopic third ventriculostomy in children. Childs Nerv Syst.

[42] Kulkarni AV, Riva-Cambrin J, Holubkov R (2016). Endoscopic third ventriculostomy in children: prospective, multicenter results from the Hydrocephalus Clinical Research Network. J Neurosurg Pediatr.

[43] Hellwig D, Giordano M, Kappus C (2013). Redo third ventriculostomy. World Neurosurg.

[44] Nigri F, Telles C, Acioly MA (2010). Late obstruction of an endoscopic third ventriculostomy stoma by metastatic seeding of a recurrent medulloblastoma. J Neurosurg Pediatr.

[45] Schulz M, Spors B, Thomale UW (2015). Stented endoscopic third ventriculostomy—indications and results. Childs Nerv Syst.

[46] Amini A, Schmidt RH (2005). Endoscopic third ventriculostomy in adult patients. Neurosurg Focus.

[47] Etus V, Kahilogullari G, Gokbel A, Genc H, Guler TM, Ozgural O, Unlu A (2021). Repeat endoscopic third ventriculostomy success rate according to ventriculostoma closure patterns in children. Childs Nerv Syst.

[48] Rahman MM, Khan SI, Khan RA, Islam R, Sarker MH (2021). Endoscopic third ventriculostomy in children: problems and surgical outcome: analysis of 34 cases. Chin Neurosurg J.

[49] Faggin R, Calderone M, Denaro L, Meneghini L, d'Avella D (2011). Long-term operative failure of endoscopic third ventriculostomy in pediatric patients: the role of cine phase-contrast MR imaging. Neurosurg Focus.

[50] Mahapatra A, Mehr S, Singh D, Tandon M, Ganjoo P, Singh H (2011). Ostomy closure and the role of repeat endoscopic third ventriculostomy (re-ETV) in failed ETV procedures. Neurol India.

[51] Peretta P, Cinalli G, Spennato P (2009). Long-term results of a second endoscopic third ventriculostomy in children: retrospective analysis of 40 cases. Neurosurgery.

[52] Kulkarni AV, Drake JM, Kestle JR, Mallucci CL, Sgouros S, Constantini S (2010). Endoscopic third ventriculostomy vs cerebrospinal fluid shunt in the treatment of hydrocephalus in children: a propensity score-adjusted analysis. Neurosurgery.

[53] Drake J, Chumas P, Kestle J (2006). Late rapid deterioration after endoscopic third ventriculostomy: additional cases and review of the literature. J Neurosurg.

